# Effectiveness of Nifurtimox in the Treatment of Chagas Disease: a Long-Term Retrospective Cohort Study in Children and Adults

**DOI:** 10.1128/aac.02021-21

**Published:** 2022-04-13

**Authors:** N. Falk, A. J. Berenstein, G. Moscatelli, S. Moroni, N. González, G. Ballering, H. Freilij, J. Altcheh

**Affiliations:** a Parasitología, Hospital de Niños Ricardo Gutierrez, Instituto Multidisciplinario de Investigación en Patologías Pediátricas (IMIPP), CONICET-GCBA, Buenos Aires, Argentina; b Instituto Multidisciplinario de Investigaciones en Patologías Pediátricas (IMIPP), CONICET-GCBA, Laboratorio de Biología Molecular, División Patología, Hospital de Niños Ricardo Gutiérrez, Buenos Aires, Argentina

**Keywords:** *Trypanosoma cruzi*, nifurtimox, children, adults, treatment effectiveness, efficacy, follow-up, treatment

## Abstract

Chagas disease (ChD), caused by Trypanosoma cruzi, has a global prevalence due to patient migration. However, despite its worldwide distribution, long-term follow-up efficacy studies with nifurtimox (NF) are scarce and have been conducted with only small numbers of patients. A retrospective study of a large cohort of ChD treated children and adults with NF. Treatment response was evaluated by clinical, parasitological, and serological after-treatment evaluation. A total of 289 patients were enrolled, of which 199 were children and 90 adults. At diagnosis, 89.6% of patients were asymptomatic. Overall, all symptomatic patients showed clinical improvement. At baseline, parasitemia was positive in 130 of 260 (50%) patients. All but one adult patient had cleared their parasitemia by the end of treatment. That patient was considered a treatment failure. Median follow-up time for children was 37.7 months, with an interquartile range of (IQR_25–75_ 12.2 to 85.3), and for adults was 14.2 months (IQR_25–75_, 1.9 to 33.8). After treatment, a decrease of T. cruzi antibodies and seroconversion were observed in 34.6% of patients. The seroconversion profile showed that, the younger the patient, the higher the rate of seroconversion (log rank test; *P* value, <0.01). At least 20% seroreduction at 1 year follow-up was observed in 33.2% of patients. Nifurtimox was highly effective for ChD treatment. Patients had excellent treatment responses with fully resolved symptoms related to acute T. cruzi infection. Clearance of parasitemia and a decrease in T. cruzi antibodies were observed as markers of treatment response. This study reinforces the importance of treating patients during childhood since the treatment response was more marked in younger subjects. (This protocol was registered at ClinicalTrials.gov under registration number NCT04274101).

## INTRODUCTION

Chagas disease (ChD), caused by Trypanosoma cruzi, currently affects over 7 million people in Latin America. Over the past few decades, ChD has spread to the rest of the world via the migration of infected people, with the majority of cases reported in Europe, North America, Australia, and Japan (1). The acute phase, usually asymptomatic, is followed by a chronic stage that can lead to an irreversible heart disease many years later (2) and causes more than 7,000 deaths yearly.

Nifurtimox (NF) and benznidazole (BZ) represent the only two available treatments to date, with no obvious difference in their effectiveness. In addition, although BZ has been more commonly used for ChD, new developments for NF have been undertaken, and a pediatric formulation has recently been released (3).

It is important to highlight that the evaluation of the therapeutic response in ChD is challenging, mainly due to prolonged persistence of Trypanosoma cruzi-specific antibodies and the lack of sensitivity of parasitological tests. This scenario calls for long-term follow-up of treated patients in order to observe negative seroconversion of conventional T. cruzi serological tests. There are few studies evaluating NF efficacy; long-term follow-up efficacy studies are currently scarce and conducted with only small numbers of patients.

There have been some concerns about the safety of NF. In a previous study, we demonstrated that NF is a safe option for ChD treatment (4). In this study, we systematically assessed the effectiveness of NF considering the same ChD cohort population, which included both children and adult patients with a long-term follow-up of more than 10 years.

## RESULTS

**FIG 1:**
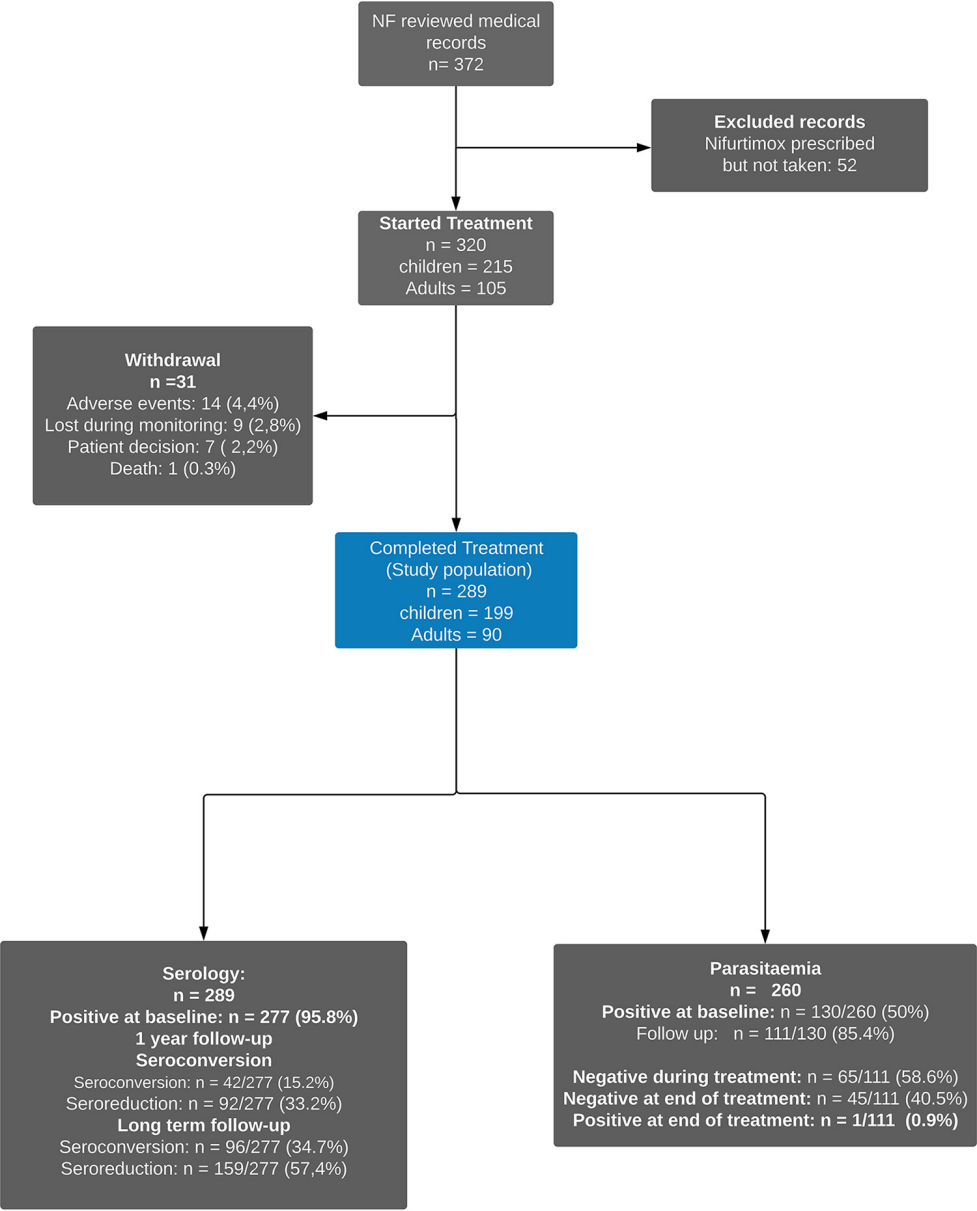
Flow diagram.

Medical records of ChD patients treated at our service were reviewed, and 372 patients who were prescribed NF were identified. After applying selection criteria, 289 patients (199 children and 90 adults) were included (Fig. 1, blue box).

Demographic data are shown in Table 1. Male and female subjects were well balanced in children but not in adults, where 85.6% of subjects were female. This imbalance was due to the fact that most of the adult patients were mothers of children assisted in our service. The route of infection was congenital in 121 (41.8%) subjects, undetermined in 136 (47.1%) subjects, vector borne in 30 (10.4%) subjects, and by blood transfusion in 2 (0.7%) subjects.

**TABLE 1:** Demographic data^*a*^

Demographic	No. of patients (%) (0–8 mo); *n* = 53	No. of patients (%) (8 mo–2 yrs); *n* = 41	No. of patients (%) (2–7 yrs); *n* = 41	No. of patients (%) (7–12 yrs); *n* = 35	No. of patients (%) (12–18 yrs); *n* = 29	No. of patients (%) ≥18 yrs; *n* = 90	Total no. of patients (%); *n* = 289
Gender							
Female	30 (56.6)	17 (41.5)	17 (41.5)	17 (48.6)	20 (69.0)	77 (85.6)	178 (61.6)
Male	23 (43.4)	24 (58.5)	24 (58.5)	18 (51.4)	9 (31.0)	13 (14.4)	111 (38.4)
Age (mo)							
Median [Q1, Q3]	2.00 [1.00, 4.00]	13.0 [10.0, 15.0]	48.0 [32.0, 60.0]	120 [96.0, 120]	168 [156, 192]	414 [351, 456]	96 [12, 336]
Mean (SD)	2.66 (1.74)	13.0 (3.55)	45.5 (14.6)	110 (17.1)	172 (21.0)	412 (88.3)	168 (179)
Min-max	1.00–7.00	8.00–19.0	24.0–72.0	84.0–132	144–215	228–684	1.00–684
Route of infection							
Vector borne	1 (1.9)	3 (7.3)	9 (22.0)	6 (17.1)	3 (10.3)	8 (8.9)	30 (10.4)
Congenital	51 (96.2)	34 (82.9)	16 (39.0)	7 (20.0)	5 (17.2)	8 (8.9)	121 (41.9)
Blood transfusion	0	0	1 (2.4)	0	0	1 (1.1)	2 (0.7)
Undetermined	1 (1.9)	4 (9.8)	15 (36.6)	22 (62.9)	21 (72.4)	73 (81.1)	136 (47.1)

a Age group notation uses a parenthesis when the age limit is not included in the group and a squared bracket when the age limit is included (see section Study design and population).

Treatment was as follows: median NF dose for children was 11 mg/kg of body weight per day, with an interquartile range of (IQR_25–75_, 10 to 12 mg/kg) in 2 divided doses (*n* = 139) or 3 divided doses (*n* = 60) with a median length of 62 days (IQR_25–75_, 60.5 to 73 days). The mean NF dose for adult patients was 9 mg/kg (IQR, 8.2 to 9.7 mg/kg) in 2 (*n* = 46) or 3 divided doses (*n* = 44) with a median length of 30 days (IQR_25–75_, 29 to 32 days). Good treatment compliance was observed, and treatment was well-tolerated, since the vast majority of observed adverse drug reactions (ADRs) were mild. Details of observed ADRs were described in depth in our previous publication (4).

Mean follow-up time for children was 37.7 months (IQR_25–75_, 12.2 to 85.3 months). Retention of pediatric patients during long-term follow-up was 84% at 5 months after treatment, 75% at 12 months, 64% at 24 months, 52% at 36 months, and 36% at 60 months. It should be noted that the treatment of adults, implemented from 2008 in our service, yielded shorter follow-up times than children with a median of 14.2 months (IQR_25–75_, 1.9 to 33.8 months). Adult retention was 71% at 5 months after treatment, 53% at 12 months, 32% at 24 months, 21% at 36 months, and only 7% at 60 months.

### Clinical.

Overall, most of the patients (259/289 [89.6%]) were asymptomatic, and only 30/289 (10.4%) were symptomatic. Taking into account the route of infection, 12/30 (40%) of those infected by the vector-borne route were symptomatic. The most frequent symptom was the ocular chagoma in 11/12 cases (Table 2). Patients infected by blood transfusion (*n* = 2) and undetermined causes (*n* = 136) were asymptomatic. A total of 18/121 (14.9%) patients infected by the congenital route were symptomatic. These cases were observed only in infants younger than 2 years, and the organ most affected was the liver (Table 2). A clinical improvement was observed in all symptomatic patients during treatment without clinical relapses during follow-up. An infant coinfected with T. cruzi and HIV by the transplacental route developed encephalitis and myocarditis. This patient died during NF treatment due to respiratory complications unrelated to NF treatment.

**TABLE 2:** Clinical finding in symptomatic patients (vector borne and congenital)^*a*^

Route of infection/clinical finding	No. of patients (%)
Vector borne	*n* = 30
Clinical examination	
Symptomatic	12 (40.0)
Symptoms	
Ocular chagoma	11 (36.7)
Myocarditis	2 (6.7)
Limbs chagoma	1 (3.3)
Generalized edema	1 (3.3)
Hepatomegaly	1 (3.3)
	1 (3.3)
Congenital	*n* = 121
Clinical examination	
Symptomatic	18 (14.9)
Symptoms	
Hepatomegaly	12 (9.9)
Jaundice	3 (2.5)
Myocarditis	5 (4.1)
Splenomegaly	2 (1.7)
Generalized edema	1 (0.8)
Petechiae	1 (0.8)
Tachycardia	1 (0.8)
Respiratory distress	1 (0.8)
Pneumonia	1 (0.8)
Hepatitis	1 (0.8)
Cholelithiasis	1 (0.8)
Seizures	1 (0.8)

a All patients infected by blood transfusion and undetermined routes were asymptomatic. Note that a patient may present more than one symptom.

### Cardiological assessment.

Electrocardiogram (ECG) was conducted at baseline in 219/289 (75.8%) patients, and 66/219 (30.1%) of these patients were evaluated every year for after-treatment follow-up. At baseline, 18/219 (8.2%) displayed alterations in their ECG, but only 11/18 (61.1%) were of clinical significance. A total of 7/18 (38.9%) patients, mainly infected by the vector-borne route, showed clinical and ECG alterations related to acute myocarditis. These patients presented clinical improvement during treatment and normalization of the ECG during follow-up. Four of these 18 patients (22.2%) showed a left bundle branch block probably related to ChD, and the remaining 7/18 (38.9%) patients showed alterations considered unrelated to ChD (4 incomplete right bundle branch blocks [IRBBB], 1 long QT, 1 sinoatrial block, and 1 isolated ventricular extrasystole). During follow-up, 2 patients with normal ECG at baseline showed ECG alterations (one patient presented an IRBBB, and the other isolated ventricular extrasystoles with an adequate chronotropic range) but without clinical significance.

### Parasitology.

At baseline, parasitemia was positive in 130/260 (50%) patients using different tests (Fig. 1; see also Fig. S1 in the supplemental material), and 111/130 (85.4%) patients had parasitological follow-up. Overall, 110/111 (99.1%) cleared parasitemia at the end of treatment. In 67/111 patients, parasitemia was evaluated during treatment, and 65/67 (97%) of them became negative during treatment. Of note, those 110/111 patients showed a persistently negative parasitemia during follow-up.

Only 1/111 (0.9%) patients (an adult female) showed positive parasitemia at the end of treatment and without a decrease in T. cruzi antibodies during follow-up. This was defined as treatment failure, and a second course of treatment with benznidazole was prescribed with a subsequent good treatment response.

### Serology.

At baseline, serology was positive in 277/289 (95.8%) evaluated patients. We observed 12 patients showing negative serology but positive parasitemia using a direct parasitological test confirming acute T. cruzi infection (see Fig. S2 in the supplemental material). These patients were in the early acute phase of the infection with high parasitemia. In 11/12 patients younger than 8 months, congenital infections were confirmed. The remaining patient, who was 17 months old, was an acute vector-borne case.

In 248/277 patients, parasitemia was tested at baseline, and in 118/277 (42.6%) patients, parasitemia was positive (Fig. S2).

### (i) Seroconversion.

After treatment follow-up, seroconversion was observed in 96/277 (34.6%) patients, 95/96(98.9%) of whom were children (Table 3). All patients reaching seroconversion had previously cleared parasitological results during follow-up.

**TABLE 3:** Kaplan-Meier summary analysis for median time to seroconversion and seroreduction events (see Fig. 2)^*a*^

Serconversion/seroreduction	No. of patients (%) (0–8 mo); *n* = 43	No. of patients (%) (8 mos–2 yr); *n* = 39	No. of patients (%) (2–7 yrs); *n* = 41	No. of patients (%) (7–12 yrs); *n* = 35	No. of patients (%) (12–18 yrs); *n* = 29	No. of patients (%) ≥18 yrs; *n* = 90	Total no.; *n* = 277
Seroconversion (%)	34 (79)	26 (66.6)	22 (53.6)	9 (25.7)	4 (13.8)	1 (1.1)	96 (34.7)
Median time to seroconversion (mo) [95% CI]	5.6 [4.1–10.6]	26.4 [13.4–42.1]	69.7 [45.8–142]	144.1 [144–nd]	142.5 [58.7–nd]		
Seroreduction (%)	38 (88.3)	32 (82.0)	29 (70.7)	22 (62.8)	20 (68.9)	18 (20.0)	159 (57.4)
Median time to seroreduction (mo) [95% CI]	3.8 [2.8–5.6]	8.4 [5.8–13.6]	31.2 [13.8–55.1]	37.8 [20.6–109]	15.0 [8.4–147]	31.9 [28.9–nd]	

a The observed differences in median times to seroconversion and seroreduction among age groups was statistically significant (*P* = <0.01; log rank test). Only patients with positive serology at baseline were included. All summary statistics were obtained by means of the “survival” R package. Confidence intervals (CI) were computed using a logarithmic transformation of the survival function. Upper confidence level (UCL95) of median times requires to be computed at least one time point in the survival curve displaying a UCL95 estimation under 0.5 survival probability. Older age groups presenting a few events often do not satisfy that criterion. In such cases, the 95% CI depicts an “nd” symbol (no data).

**FIG 2:**
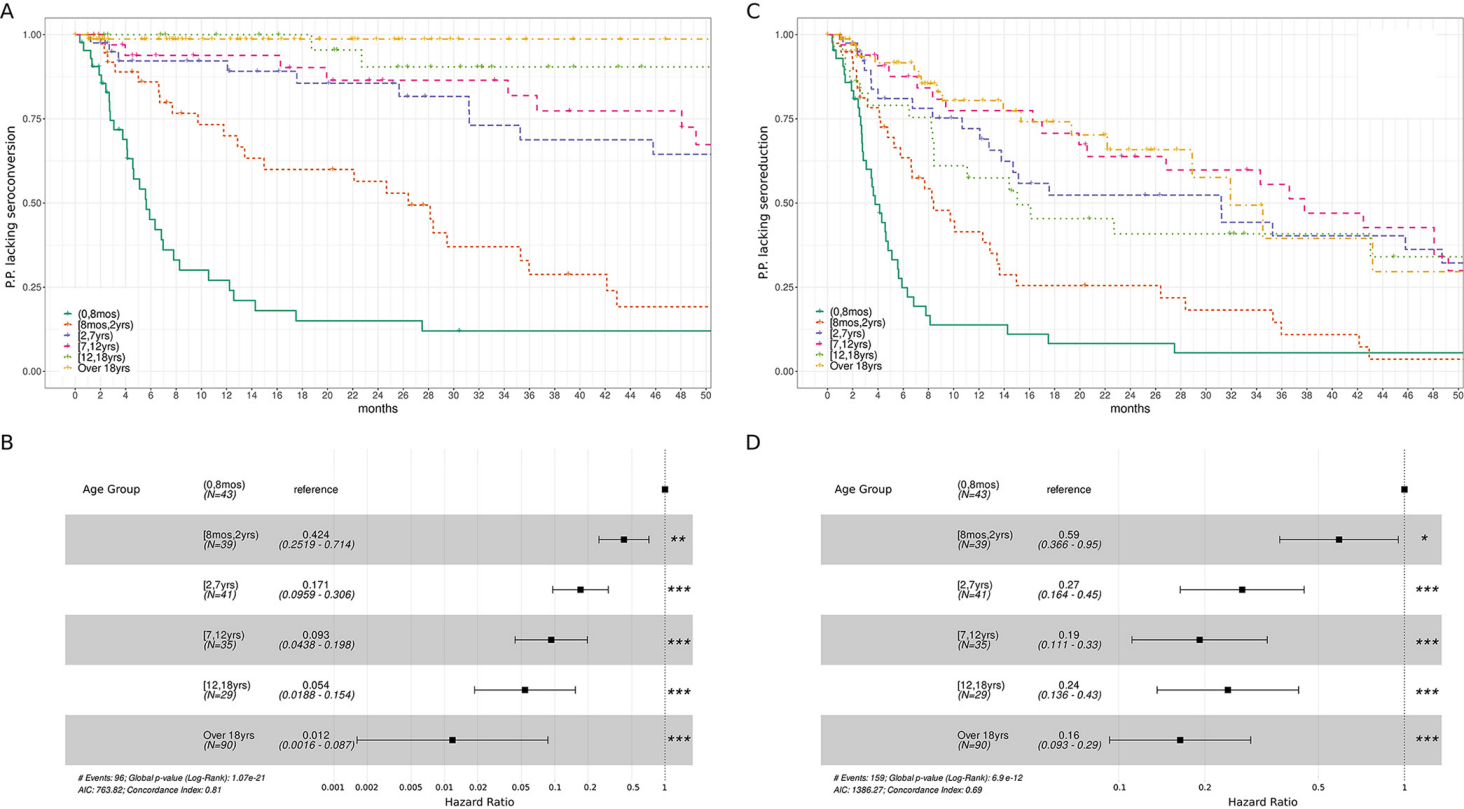
(A to C) Kaplan-Meier survival curves for seroconversion profile (A) and seroreduction profile (C) analysis stratified by age group. Ordinates depict the proportion of patients (P.P.) lacking seroconversion (A) or seroreduction (C). Only patients with positive serology at baseline 277/289 (95.8%) were included in the analysis. Of note, patients in the 0 to 8 months group have the fastest seroconversion rate. (B to D) Cox regression models for seroconversion (B) and seroreduction (D) analysis. In both cases, the infant age groups (0 to 8 months) were considered as reference for hazard ratio computing. Significance *P* values resulting from comparing HR among the considered reference are depicted according to the following: *, <0.05; **, <0.01; ***, <0.001. The whole analysis was performed by using the “survival” R package version 3.2-7 (29).

For patients with reactive serology at baseline (*n* = 277), a Kaplan-Meier survival analysis stratified by age groups was conducted (Fig. 2A). The median time of seroconversion increased with age and showed differences between age groups (Table 3; *P* = <0.01; log rank test). A Cox regression model showed that the hazard of seroconversion decreases as the age of the groups increases (Fig. 2B).

### (ii) Seroreduction.

A total of 159/277 (57.4%) patients showed seroreduction from baseline antibody titers during long-term follow-up. In children, seroreduction was observed in 141/159 (88.7%) patients (Table 3).

A Kaplan-Meier survival analysis stratified by age groups was conducted (Fig. 2C). Median seroreduction times showed differences between age groups and increased with age (Table 3) (*P* = <0.01; log rank test). A Cox regression model showed that the hazard of seroreduction decreases as the age of the groups increases until 2 to 6 years, after which, all hazard ratio intervals overlap (Fig. 2D).

We also analyzed the rates of seroreduction by age group at a 1-year follow-up (Table 4). Overall, at the 1-year follow-up 92/277 (33.2%) patients showed seroreduction. In long-term follow-up, antibody reduction increased over time as a marker of treatment response. In 10/90 (11.1%) evaluated adults, seroreduction was observed at the 1-year follow-up, although only 1 patient seroconverted during follow-up.

**TABLE 4:** Analysis of seroconversion and seroreduction at 1 year follow-up^*a*^

Age group	No. (seropositive at baseline)	No. (1-yr follow-up)	Seroconversion (%)	Seroreduction (%)
(0–7 mo)	43	38	26 (60.5)	33 (76.7)
(8 mos**–**2 yrs)	39	33	10 (25.6)	20 (51.2)
(2**–**6 yrs)	41	33	3 (7.3)	10 (24.4)
(7**–**11 yrs)	35	30	2 (5.7)	7 (20.0)
(12**–**17 yrs)	29	27	0 (0)	12 (41.4)
(Over 18 yrs)	90	38	1 (1.1)	10 (11.1)
Total	277	199	42 (15.2)	92 (33.2)

a Only patients with positive serology at baseline who were studied for at least 1 year after diagnosis were described. Percentages were computed in a conservative way by using the number of patients positive at baseline as denominator. In this way, we avoid any possible bias due to loss of patient follow-up.

Interestingly, the patient who did not clear parasitemia and was considered a treatment failure did not show any reduction from baseline antibodies.

### (iii) Stratified serological analysis by parasitological baseline profile.

**FIG 3:**
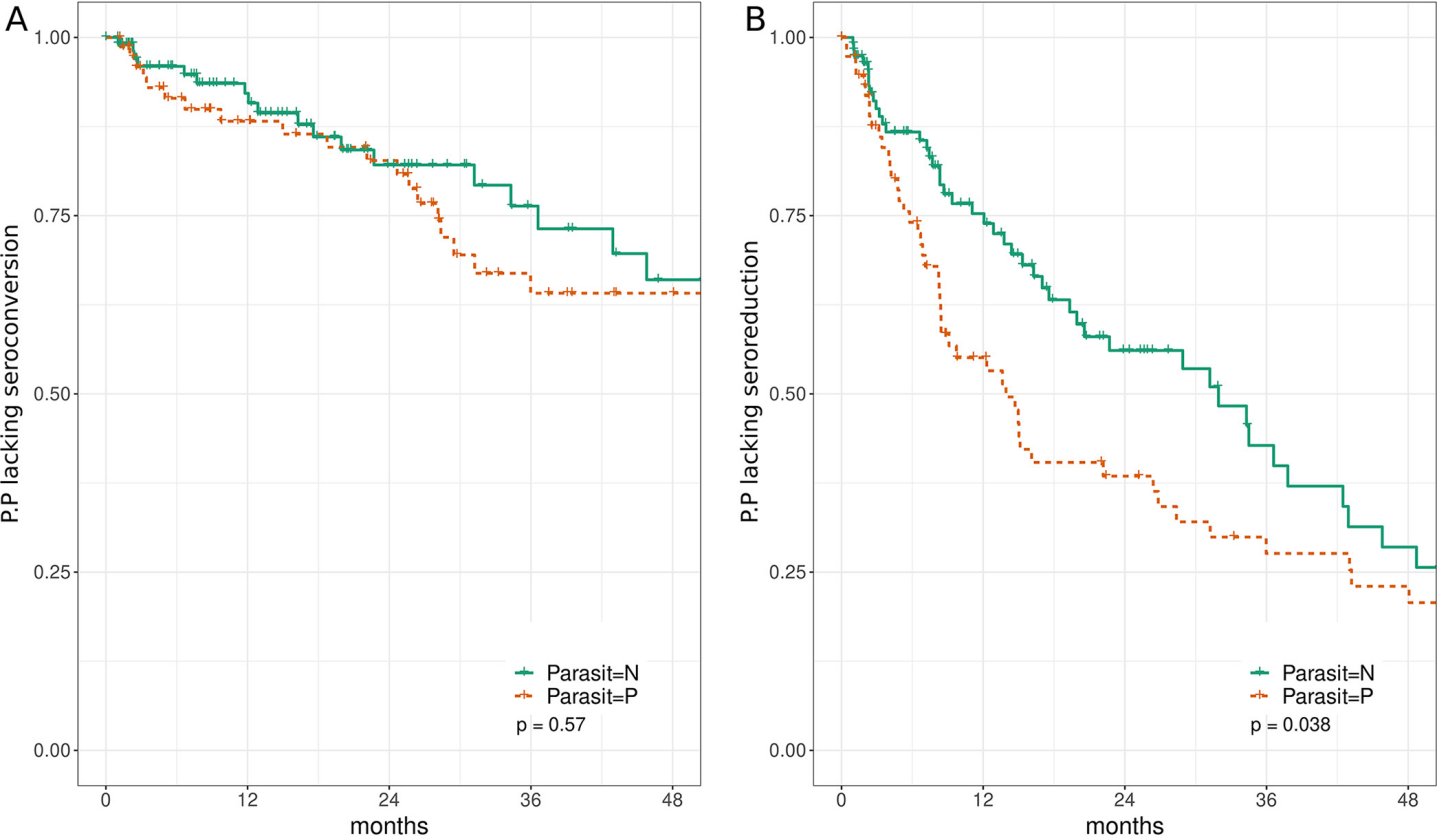
Kaplan-Meier curves of seroconversion (A) and seroreduction (B) stratified by parasitological results at baseline. Ordinates depict the proportion of patients (P.P.) lacking seroconversion (A) or seroreduction (B). Only patients with positive serology at baseline and a performed parasitological test were included in the analysis. Patients in the infant group (0 to 8 months) were excluded from this analysis in order to avoid a bias caused by the diagnosis criteria (see main text). Patients presenting a negative parasitological result at baseline (*n* = 130) were plotted in green (Parasit = N) and patients presenting a positive parasitological result (*n* = 76) at baseline were plotted in red (Parasit = P).

Here, taking advantage of the considerable number of patients having both serological and parasitological tests, we aimed to investigate if patients with positive parasitemia at baseline had a different serological after treatment response profile over time than those with negative baseline parasitemia. To this end, we stratified patients according to their parasitological result at baseline. Those younger than 8 months were excluded, since all were parasitemia positive due to the diagnosis criteria. For patients older than 8 months, a Kaplan-Meier analysis of seroconversion and seroreduction profiles was performed (Fig. 3A and B). No differences were found regarding seroconversion profiles, but a difference could be appreciated when seroreduction was considered (log rank test; *P* value = 0.038). We repeated the seroreduction analysis separately for each age group to decouple it as a potential confounder (see Fig. S3 and Table S1 in the supplemental material). In that subanalysis, no major differences were observed between the seroreduction profiles inside each age group: curves overlap almost in all cases and no comparison yielded statistically significant results. In summary, the observed differences in seroreduction profiles between parasitemia-positive and parasitemia-negative patients shown in Fig. 3 cannot be disassociated in our study from the age group.

## DISCUSSION

Long-term follow-up efficacy studies with NF are scarce, and the available information is based on relatively old studies of individual cases or small samples. In addition, reports vary substantially due to diverse sampling design and quality of the studies.

In this study, we systematically assessed the effectiveness of NF in a large ChD cohort, including both children and adult patients. To the best of our knowledge, this work is one of the largest studies evaluating the effectiveness of NF with long-term follow-up of more than 10 years.

At diagnosis, few patients were symptomatic. Most of them were vector-borne cases where the diagnosis was suspected due to clinical symptoms. In contrast, patients infected by the congenital route were mainly asymptomatic as described previously (5). A good clinical treatment effect was observed since symptomatic patients recovered without clinical relapse during follow-up.

Controversy remains regarding the benefits of chemotherapy for ChD in asymptomatic patients, based on the difficulty in defining treatment response and in detecting parasitological clearance after treatment. In this regard, seroconversion is considered a marker of parasitological treatment response (6, 7). However, negative seroconversion of T. cruzi antibodies is usually only observed several years after treatment in patients in the late chronic phase, even when parasitological methods are consistently negative. This is probably due to the persistence of immunological memory rather than a lack of treatment response since the majority of treated patients showed persistent negative T. cruzi PCR.

Furthermore, our group (8) and other authors (9, 10) have interpreted accelerated decay of T. cruzi antibodies, combined with persistently cleared parasitological tests, as indicative of a treatment response, as was observed in the present study. A decrease in antibody titers with clearance of T. cruzi in blood seems to precede complete seroconversion and may indicate an evolution toward serologic cure. It is important to note that seroreduction was observed with statistically significant differences within the different age groups. The time to reach seroconversion appears to be inversely related to the duration of infection. Younger age groups showed an earlier response and a higher probability of seroconversion after treatment. Several studies showed that in untreated patients parasitemia and antibody titers remain stable in long-term follow-up (11). It would appear therefore that clearance of parasitemia and seroreduction of T. cruzi antibodies are a useful predictor of future negative seroconversion.

The need for such long-term follow-up of treated patients with the use of conventional serology has led to the evaluation of alternative techniques as potential treatment markers. PCR is a sensitive and specific method to detect parasitemia in newborns and infants and has also shown good results for the assessment of therapy failure in chronic ChD (12–14). A positive result clearly indicates failure to eliminate the parasite (15). In the present study, a considerable proportion of treated patients (58.6%) (see Fig. 1) showed parasitological clearance during the early stages of treatment, suggesting the possibility of implementing shorter treatment regimens. However, since PCR assays could have variations in sensitivity and reproducibility, further studies to standardize PCR technique would be necessary to confirm this observation. Almost all remaining patients showed late parasitemia clearance, and it is reasonable to attribute this to the fact that parasitemia was not evaluated throughout all visits, due to lack of patient attendance rather than a partial treatment response. Relatedly, recent studies have also investigated alternative regimens, with a shorter duration and/or lower doses to improve treatment tolerability and adherence (3, 8, 16). It is important to highlight that adult patients in the present study received a 30-day treatment regimen with good treatment results.

In accordance with previous literature (2, 8), our results also showed the importance of correlating parasitological clearance with seroreduction/seroconversion of T. cruzi antibodies. Currently, a combination of at least 20% seroreduction of T. cruzi antibodies with a persistent negative PCR was used as a marker of treatment response in a recently performed pivotal clinical study (3), which our group coauthored. The threshold for seroreduction was based on the results of a randomized clinical trial conducted in pediatric patients with ChD, in which a 21% reduction in optical densities measured by conventional enzyme-linked immunosorbent assay (ELISA) was observed in patients aged 6 to 12 years treated with 60-day benznidazole at a 12-month follow-up (17). The proposed marker of treatment response (cleared parasitemia and seroreduction) was clearly observed in our study, even when it was evaluated at 1 year after treatment endpoint. In this regard, it is also interesting to highlight that in the only patient with positive parasitemia (therapeutic failure), no seroreduction was observed.

Our results show the need for a more serious debate within the scientific and clinical community about the treatment response criteria of ChD. There is a clear need for earlier serological markers of treatment response. Some of them have been proposed in recent years, such as the use of nonconventional serological techniques (18, 19). In particular, the use of specific biomarkers, like anti-α-Gal lytic antibodies known as anti-F2/3 antibodies (8, 20–22), has been evaluated. However, further studies are needed for their validation in adults as well as children. Children are the gold standard for the validation of serological tests due to the high rate of seroconversion. These new markers should allow us to evaluate the treatment response for both children and adults in a more reliable way and in a shorter time scale.

Notably, our group observed a low incidence of cardiac lesions related to ChD in treated patients (23). However, the retrospective characteristics of our study in a real-world setting do not allow a conclusive result regarding this matter. Due to ethical considerations, an untreated control group cannot be included since it is unethical to withhold ChD treatment from children.

In conclusion, our study increases the understanding of the effects and outcomes of NF treatment in children and adults with ChD. Nifurtimox-induced antiparasitic clearance in both children and adults reinforces the fact that T. cruzi infection is curable if treatment is initiated soon after infection. Further studies are needed in order to evaluate the long-term clinical effect of the clearance of parasitemia to prevent the development of cardiac lesions.

## MATERIALS AND METHODS

### Study design and population.

This is a retrospective age-stratified cohort study to assess effectiveness over a long-term follow-up period of oral administration of NF in subjects with ChD. Patients treated and followed up at the Parasitology and Chagas service, Hospital de Niños “Ricardo Gutiérrez,” Buenos Aires, Argentina from January 1980 to July 2019 were included in the study.

### (i) Inclusion criteria.

Patients with at least 60 days of treatment (children) or 30 days of treatment (adults) with NF were included.

### (ii) ChD diagnostic criteria.

For infants younger than 8 months, diagnostic criteria included direct observation of T. cruzi by a parasitological concentration method as follows: microhematocrit test (MH) or xenodiagnosis (XD); for patients older than 8 months, 2 reactive serological tests by enzyme-linked immunosorbent assay (ELISA) (Wiener manufacturer) and indirect hemagglutination (IHA) or direct agglutination (DA) (Polychaco or Wiener, manufacturers) were required.

### (iii) Patient stratification.

Patients were stratified according to the following age groups: (0 to 8 months), (8 months to 2 years), (2 to 7 years), (7 to 12 years), (12 to 18 years), and adults (18 years or older). The notation employed here uses a parenthesis when the age limit is not included in the group and a squared bracket when the age limit is included. For example, a child aged 1 yr and 364 days would be included in the (8 months to 2 years) group, while a patient aged 2 years and 0 days would be included in the (2 to 7 years) group.

### Treatment.

Nifurtimox (120-mg tablets; Bayer) was prescribed in doses of 10 to 15 mg/kg per day divided into two or three daily doses for 60 to 90 days for infants and children, and 8 to 10 mg/kg for 30 days for adults, according to national guidelines. Enrollment of children started in January 1980 and for adults in July 2008. Treatment was considered complete when patients took the medication for at least 60 days for children and adolescents and 30 days for adults. Safety/tolerability of NF and adverse drug reaction (ADR) profiles, in this population, were evaluated and described by our group in a previous study (4).

### (i) Follow-up.

Baseline data values were obtained at the beginning of the treatment. Visits were carried out at 7, 30, or 60 days and, at the end of treatment, every 3 months during the first year posttreatment and every 6 to 12 months thereafter.

### (ii) Serology.

T. cruzi serology, by conventional T. cruzi ELISA, DA or IHA, was carried out at every visit. Serological results by ELISA were expressed as the ratio between the optical density (OD) value recorded for each serum sample and the cutoff value of the assay (R = OD sample/OD cutoff). IHA and DA results were expressed as an inverse log_2_ scale. ELISAs have been performed from 1995 onwards for diagnoses and for treatment follow-up.

Seroreduction was defined as a decline of at least 20% in T. cruzi antibodies titers from baseline. This was measured by ELISA and expressed as a ratio (OD sample/OD cutoff) from baseline. For IHA and DA (semiquantitative tests), a 20% decay in the log_2_ of the number of dilutions was used.

Seroconversion was defined as the event in which all performed serological tests became negative during follow-up.

### (iii) Parasitemia.

In infants younger than 8 months, MH (24) or XD (25) was performed for diagnosis and weekly until they became negative. Since 1999, the detection of T. cruzi DNA has been carried out in blood by a conventional PCR as a parasitological test (26). Since 2009, a multiplex quantitative PCR (qPCR) assay has been used to quantify the T. cruzi nuclear satellite DNA (27).

For subjects older than 8 months, parasitological tests at baseline were not systematically carried out, since diagnosis was based on serological tests.

### (iv) Treatment response criteria.

Clearance of parasitemia and seroconversion/seroreduction of conventional T. cruzi serology in at least 2 after-treatment samples during long-term follow-up. Treatment failure was defined as T. cruzi detection by parasitological tests (MH, XD, or PCR) after treatment.

### Clinical evaluation.

Clinical checkup was performed at diagnosis and at every visit.

### Data collection.

Data were collected from medical records of treated patients and stored in an access clinical database (ACD) designed for this study. All individual data sets were anonymized.

### Statistical analysis.

Continuous variables were expressed with the mean and median, as applicable, with the corresponding standard deviation or interquartile range. They were analyzed using the Student’s *t* test or Wilcoxon signed-rank test. Categorical variables were expressed in percentages. They were analyzed using the chi-square test with Fisher's correction.

The rate of seroconversion and seroreduction was estimated. Time to reach seroreduction and seroconversion was described using Kaplan-Meier survival analysis. Comparison among age groups was evaluated using log rank test and Cox regression when applied. *P* values, adjusted by false discovery rate (Benjamini-Hochberg procedure) with *P* < 0.05, were considered statistically significant. The R statistical software was used for all analysis (28). Kaplan-Meier analysis and Cox regression models were performed taking advantage of “survival” R library version 3.2-7 (29).

Study protocol was approved by the Research and Teaching Committee and the Bioethics Committee of the Buenos Aires Children’s Hospital “Ricardo Gutierrez.”
