# Supplementary material for: Effectiveness of Nifurtimox in the Treatment of Chagas Disease: a Long-Term Retrospective Cohort Study in Children and Adults
Source: Antimicrob Agents Chemother. 2022 Apr 13;66(5):e02021-21. doi: 10.1128/aac.02021-21 (PMC9112880; doi:10.1128/aac.02021-21)
Supplement: Supplemental file 1 — Fig. S1 to S3 and Table S1. Download aac.02021-21-s0001.pdf, PDF file, 0.4 MB [file aac.02021-21-s0001.pdf]

Supplementary Material

Supplementary figures

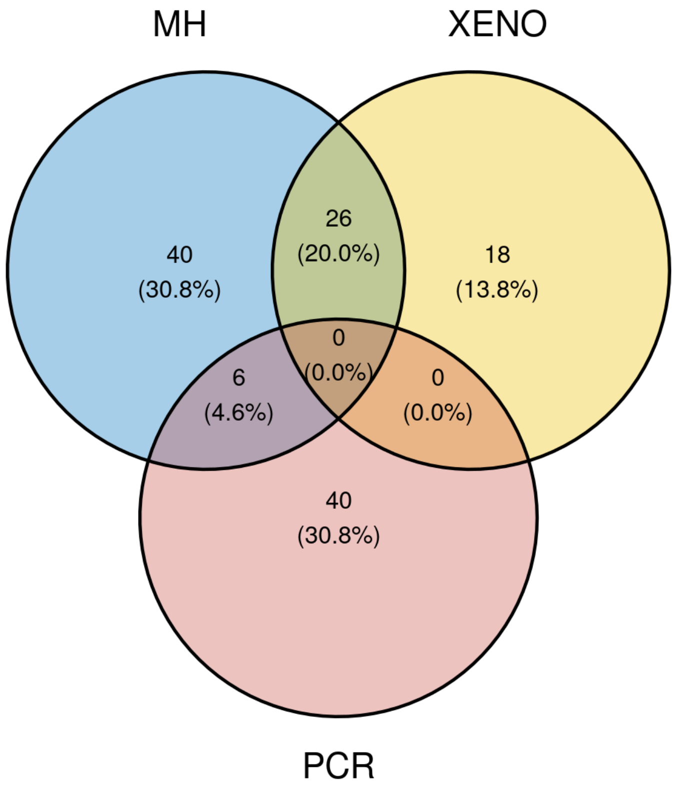

Supplementary Figure 1: Venn diagram depicting parasitological techniques used for parasitological testing at baseline. The diagram shows the relationship between MH, XD and PCR for those patients with at least one positive result obtained by any parasitological technique.

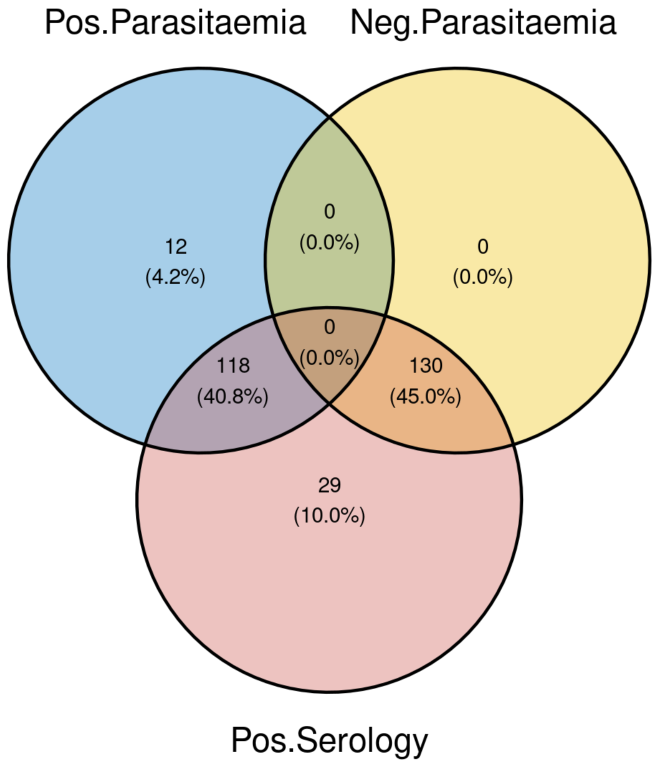

Supplementary Figure 2: Venn diagram depicting relationship between serological and parasitological testing at baseline for the 289 described patients of the study.

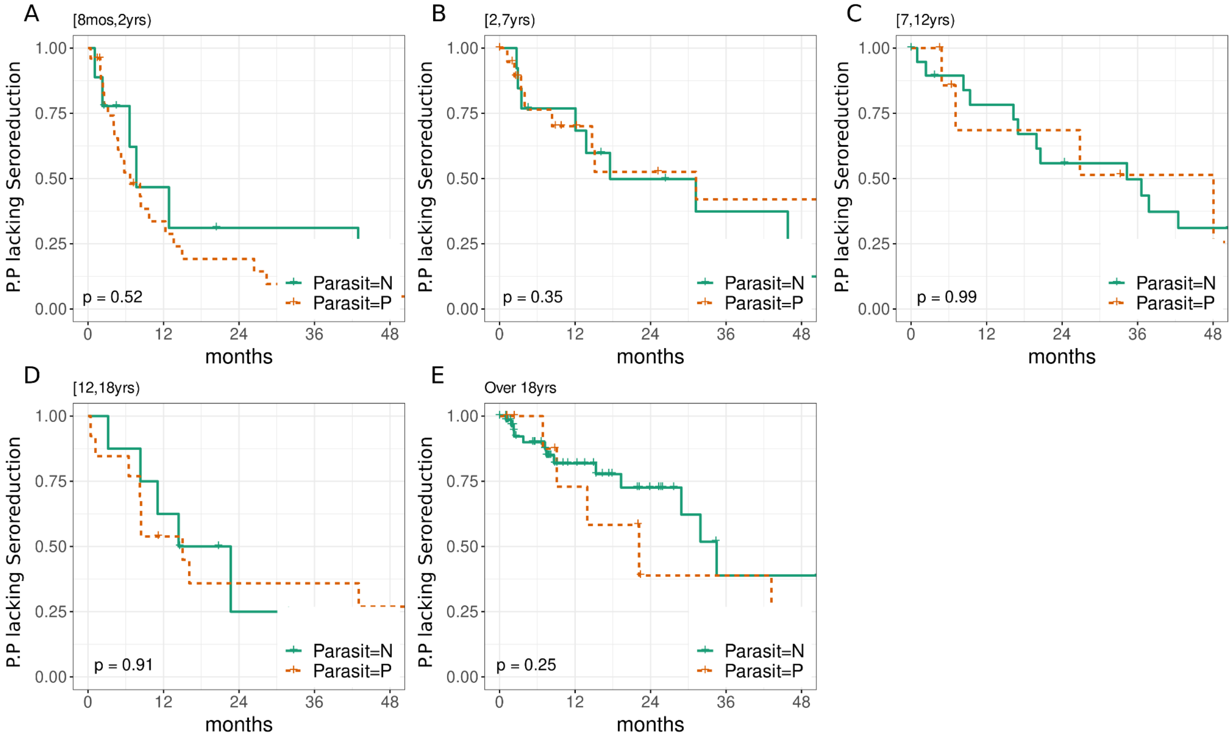

Supplementary Figure 3: Kaplan Meier curves for seroreduction stratified by age group (panels A-E) and parasitological results at baseline (green and red curves). Ordinates refers to the Proportion of Patients (P.P) lacking Seroreduction. Only patients with positive serology at baseline and a performed parasitological test were included in the analysis. Patients in the infant group (0, 8mos) were excluded from this analysis in order to avoid a bias caused by the diagnosis criteria (see main text). Patients presenting a negative parasitological result at baseline (n = 130) were plotted in green (Parasit =N) and patients presenting a positive parasitological result (n = 76) at baseline were plotted in red (Parasit =P)

Supplementary Tables

Supplementary table 1. Parasitological tests performed at baseline stratified by age group. Positive and Negative results are depicted in each column.

	neg(%)	Pos (%)
[0,8mos)	0 (0)	52 (100)
[8mos,2yrs)	9(25)	27 (75)
[2yrs,7yrs)	13 (39.4)	20 (60.6)
[7yrs,12yrs)	21 (72.4)	8 (27.6)
[12yrs,18yrs)	8 (38.1)	13 (61.9)
≥ 18yrs	79 (88.8)	10 (11.2)
